# Cytomegalovirus (CMV) Colitis Complicated by a Stricture in an Immunocompetent Adult

**DOI:** 10.7759/cureus.108452

**Published:** 2026-05-07

**Authors:** David S Pinto, Kelsy N Larios, Inderpreet Saini

**Affiliations:** 1 Medicine, David Geffen School of Medicine at UCLA, Los Angeles, USA; 2 Internal Medicine, David Geffen School of Medicine at UCLA, Los Angeles, USA

**Keywords:** atypical presentation, colonic stricture, cytomegalovirus (cmv) infection, delay in diagnosis, immunocompetent adult

## Abstract

Although cytomegalovirus (CMV) seropositivity is common in the general population, primary CMV infection in immunocompetent adults is usually asymptomatic or causes only a mild mononucleosis-like illness, and severe symptomatic or tissue-invasive disease is uncommon. CMV colitis may present with nonspecific symptoms, delaying recognition, particularly in immunocompetent patients in whom clinical suspicion is often low. Delayed diagnosis may allow progression to serious complications, including gastrointestinal bleeding, perforation, toxic megacolon, and, less commonly, stricture formation. We report a case of CMV colitis in an immunocompetent woman presenting with a colonic stricture initially presumed to be malignant rather than with the more typical features of abdominal pain, diarrhea, fever, and hematochezia. This case emphasizes the diagnostic challenge of CMV colitis in patients without classic risk factors, particularly when it mimics colonic malignancy and requires tissue diagnosis because noninvasive testing may not reliably exclude disease.

## Introduction

Cytomegalovirus (CMV) is an enveloped double-stranded DNA herpesvirus and a ubiquitous infection worldwide. It is transmitted through infected bodily fluids, vertical transmission, blood products, and solid organ or hematopoietic transplantation, contributing to its high seroprevalence despite the relative rarity of severe symptomatic disease in immunocompetent individuals [1-3]. Among immunocompetent adults, CMV seroprevalence varies widely according to geographic, socioeconomic, and age-related factors, ranging from approximately 40% to 70% in North America and parts of Western Europe to more than 90% in many regions of Africa, Asia, and Latin America [4,5]. After primary infection, CMV establishes lifelong latency predominantly within myeloid progenitor cells and monocytes, a state maintained in part by latency-associated viral genes such as UL138 [6]. Reactivation may occur when immune surveillance is impaired, classically in advanced HIV infection with CD4+ T-cell depletion or in the setting of pharmacologic immunosuppression after transplantation or chemotherapy [3]. In ostensibly immunocompetent hosts, reactivation has also been proposed to result from transient immune dysregulation related to critical illness, sepsis, inflammatory stress, or aging [7]. Although CMV infection is common in the general population, symptomatic end-organ disease is uncommon in immunocompetent adults, and CMV colitis is a rare manifestation that occurs far more frequently in immunocompromised patients [1, 8]. The following case describes CMV colitis in an immunocompetent patient complicated by colonic stricture and illustrates the complex diagnostic evaluation required before the eventual diagnosis was established.

## Case presentation

A 72-year-old female patient with a history of hypertension presented to the emergency department with three weeks of waxing and waning right lower quadrant abdominal pain associated with nausea and documented temperatures up to 37.6°C during admission while receiving acetaminophen, as well as one week of hematochezia accompanied by dizziness and fatigue. The pain was not clearly related to meals but was variably associated with bowel movements, sometimes worsening with defecation and sometimes improving afterward. Her hemoglobin was 8.7 g/dL on admission and declined to a nadir of 7.3 g/dL on hospital day 2, likely contributing to her dizziness and fatigue. She denied chills, night sweats, unintentional weight loss, hematemesis, and melena. She also reported no prior history of inflammatory bowel disease and had never undergone a colonoscopy before this illness. Orthostatic vital signs were not documented during the admission.

Two weeks before presentation, the patient had been admitted to an outside hospital, where the CT of the abdomen and pelvis showed proximal ascending colonic wall thickening with slight adjacent fat stranding. Because the abnormality was not clearly centered on a diverticulum, focal colitis or poorly visualized diverticulitis was considered, although an underlying neoplasm could not be excluded. There was no evidence of small bowel obstruction, free air, or abscess. Colonoscopy with biopsy was also reportedly performed during that admission. Although the detailed endoscopic findings were unavailable for review, the biopsy was reportedly negative. She was referred for surgical follow-up, but did not attend before her current presentation.

**Figure 1:**
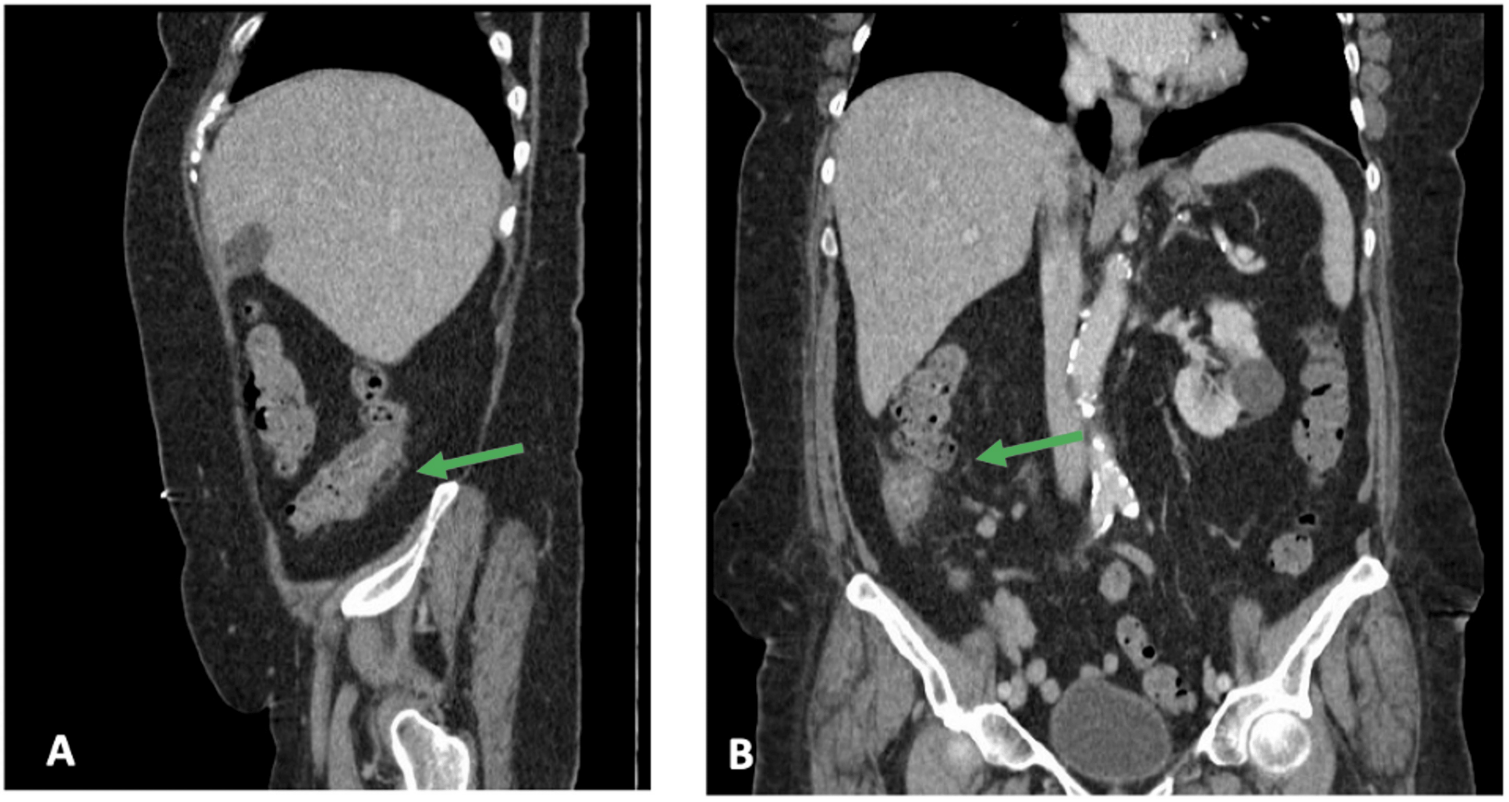
Computed tomography of the abdomen and pelvis (A) Sagittal and (B) coronal views, taken on the day of presentation at the Ronald Reagan UCLA emergency department, show mid-ascending wall thickening spanning approximately 4 cm with trace surrounding stranding (green arrow).

In the emergency department, a CT scan of the abdomen and pelvis with contrast (Figure 1) revealed a short segment mid-ascending colonic wall thickening spanning approximately 4 cm with trace surrounding stranding. Laboratory analysis revealed a hemoglobin of 8.7 g/dL (Table 1) and a normal white blood cell count with no evidence of lymphocytosis (Table 1). 

**Table 1:** Complete blood count with differential at presentation

	Patient Values	Reference value
White Blood Cell Count	5.33 x10^3^/uL	4.16-9.95 x 10^3^/uL
Red Blood Cell Count	3.24 x10^6^/uL	3.96-5.09 x 10^6^/uL
Hemoglobin	8.2 g/dL	11.6-15.2 g/dL
Hematocrit	25.7%	34.9-45.2 %
Mean Corpuscular Volume	79.3 fL	79.3 -98.6 fL
Mean Corpuscular Hemoglobin	25.3 pg	26.4-33.4 pg
MCH Concentration	31.9 g/dL	31.5- 35.5 g/dL
Red Cell Distribution Width-SD	45.1 fL	36.9-48.3 fL
Red Cell Distribution Width- DV	15.7%	11.1-15.5 %
Platelet Count, Auto	328 x10^3^/uL	143-398 x 10^3^/uL
Mean Platelet Volume	10.6 fL	9.3 – 13.0 fL
Nucleated Red Blood Cell (RBC%)	0.0%	No reference range
Absolute Nucleated RBC Count	0.00 x 10^3^/uL	0.00 – 0.00 x 10^3^/uL
Neutrophil Percent	47.6%	No reference range
Lymphocyte Percent	34.3%	No reference range
Monocyte Percent	9.9%	No reference range
Eosinophil Percent	7.3%	No reference range
Basophil Percent	0.7%	No reference range
Immature Granulocytes	0.2%	No reference range
Absolute Neutrophil Count	2.56%	No reference range
Absolute Lymphocyte Count	1.84 x10^3^/uL	1.80-6.90 x 10^3^/uL
Absolute Monocyte Count	0.53 x10^3^/uL	1.80-6.90 x 10^3^/uL
Absolute Eosinophil Count	0.39 x10^3^/uL	1.80-6.90 x 10^3^/uL
Absolute Basophil Count	0.04 x10^3^/uL	1.80-6.90 x 10^3^/uL
Absolute Immature Granulocyte Count	0.01 x10^3^/uL	1.80-6.90 x 10^3^/uL

**Figure 2:**
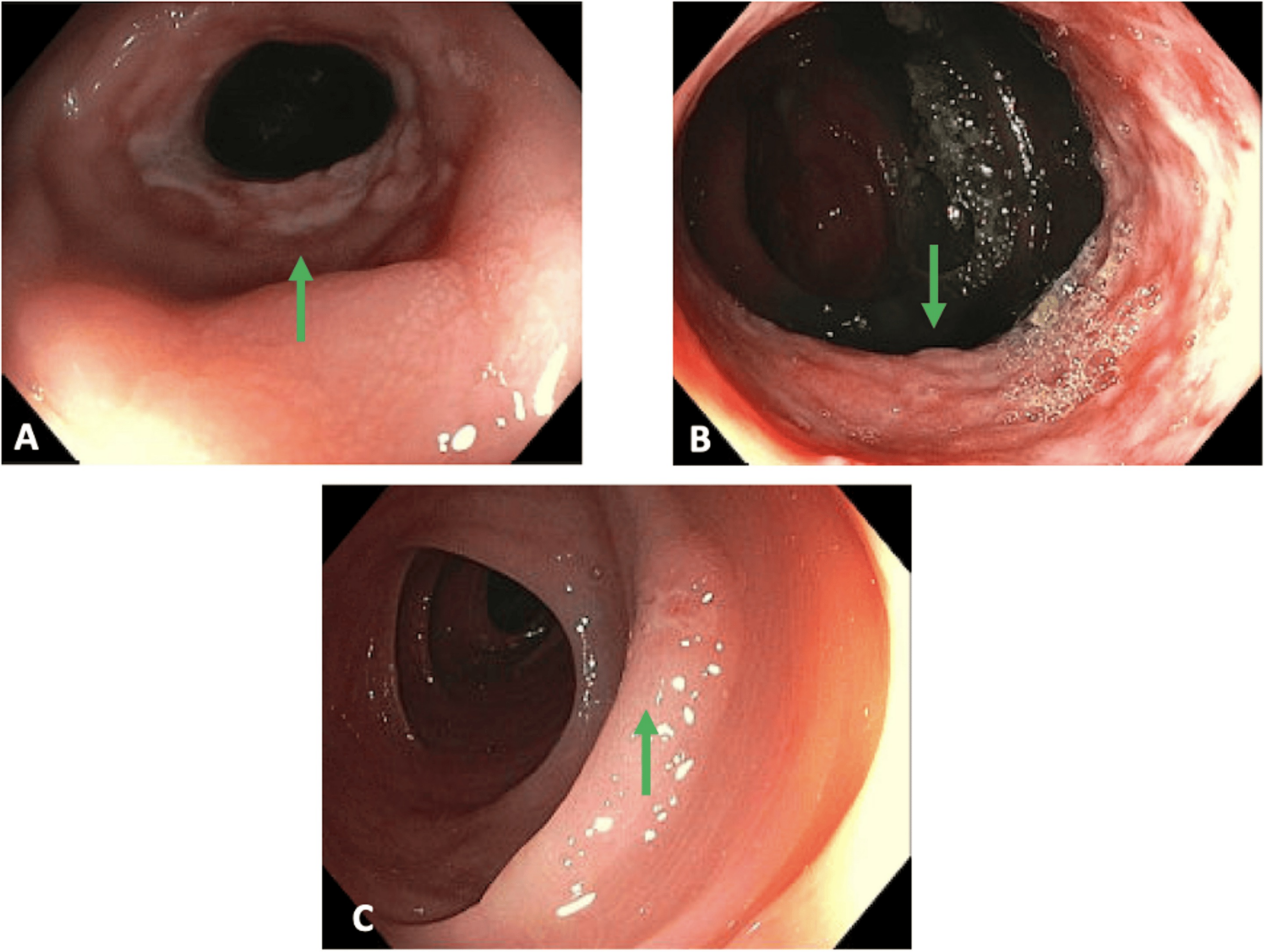
Colonoscopy images (A) Strictured ulcerated segment in the ascending colon; (B) Within the stricture segment; (C) Small area of ulceration in mid-descending colon

The patient was admitted to the medicine team for further evaluation. Gastroenterology was consulted. Initial infectious workup, not including CMV, was negative. Subsequent colonoscopy revealed a 3 cm ulcerated segment of the ascending colon with narrowing located 6-8 cm distal to the cecum and a 3-4 mm area of ulceration in the mid-descending colon (Figure 2). Both ulcerated areas were biopsied. The differential diagnosis at this time included ischemic vs infectious vs inflammatory bowel disease (IBD) vs malignancy.

**Figure 3:**
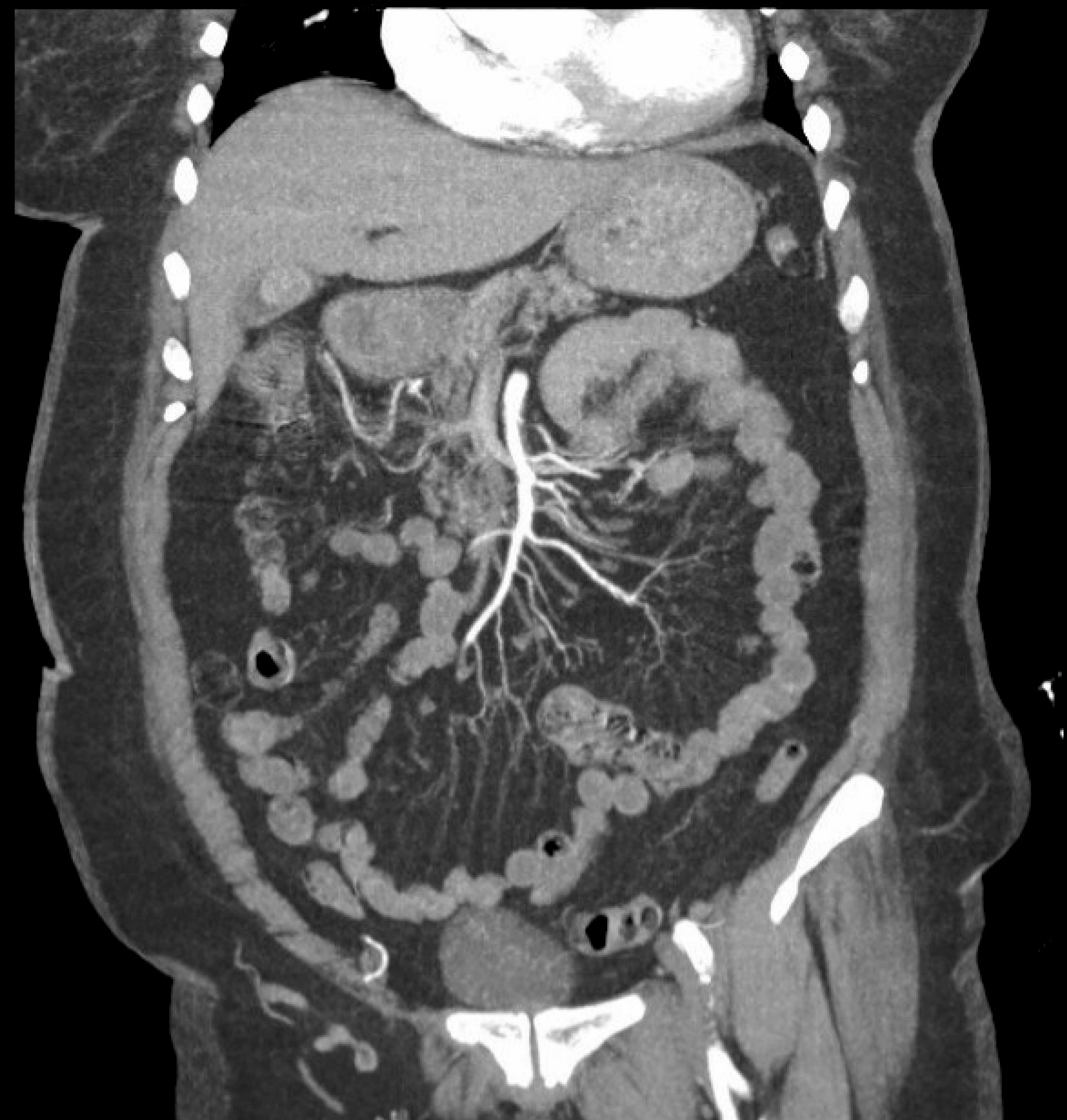
Contrast-enhanced CT angiogram of the abdomen and pelvis with contrast demonstrating a patent superior mesenteric artery (SMA) with no evidence of stenosis or occlusion.

Given the concern for ischemic colitis, a surgical consultation was obtained, and a CT angiogram of the abdomen and pelvis was performed, which revealed patent vessels without evidence of an ischemic etiology (Figure 3). At this time, serologies for IBD were collected and resulted in negative results. Throughout the hospitalization, the patient remained hemodynamically stable; however, she continued to have ongoing right lower quadrant pain, which was symptomatically managed with acetaminophen, hyoscyamine, and transdermal lidocaine. 

On day 6 of hospitalization, pathology results from the colon biopsy subsequently identified CMV IgG in the ulcerated stricture of the ascending colon. Infectious Diseases was consulted and recommended serologic testing for HIV and CMV, as well as an ophthalmology evaluation, which showed no ocular manifestations of CMV retinitis. HIV 1/2 antigen(Ag)/antibody(Ab) screen (fourth-generation) serology was nonreactive, while CMV DNA PCR was positive (Table 2). The patient was initiated on 2.5 mg intravenous ganciclovir for three days and subsequently discharged on a three-week course of valganciclovir at 450 mg daily.

**Table 2:** Virology studies ^1^Cytomegalovirus (CMV) deoxyribonucleic acid (DNA) qualitative polymerase chain reaction (qPCR), ^2^Human immunodeficiency virus (HIV) 1/2 antigen(Ag)/antibody(Ab) screen fourth-generation

	Patient Value	Reference Range
CMV DNA qPCR^1^	188 IU/mL	Not detected
HIV-1/2 Ag/Ab Screen 4^th^-Generation^2^	Nonreactive	Nonreactive

## Discussion

We present a case of CMV colitis in an immunocompetent patient that underscores the importance of maintaining a broad differential diagnosis in the evaluation of abdominal pain with colonic abnormalities. Because symptomatic CMV colitis is rare in immunocompetent individuals, its low initial suspicion, together with this patient’s atypical presentation, contributed to delayed diagnosis.

At presentation, the patient’s hematochezia and colonic wall thickening on CT raised concerns for malignancy. However, our initial differential diagnosis also included ischemic colitis, IBD, and infectious colitis. What makes this case particularly notable is the discovery of a colonic stricture on colonoscopy, an unusual finding in CMV colitis, which added complexity to the diagnostic process.

The colonic stricture observed in this patient highlights the need to consider CMV colitis when malignancy is suspected but unconfirmed. This diagnostic challenge has been described in other immunocompetent patients, including a case of CMV colitis masquerading as rectal malignancy, in which the lesion regressed on repeat colonoscopy after antiviral therapy, underscoring both the value of tissue diagnosis and the potential role of endoscopic follow-up in mass-like or stricturing presentations [9]. A similar case involved a 58-year-old male patient presenting with fever, right lower quadrant abdominal pain, vomiting, and loss of appetite. Imaging revealed colonic wall thickening and significant luminal narrowing in the ascending colon, leading to a presumptive diagnosis of colon carcinoma and subsequent right hemicolectomy. However, histopathological examination revealed multiple ulcerated lesions with granulation tissue and inflammatory cells, consistent with a CMV pseudotumor [10].

Another case highlights the need for greater attention to why immunocompetent individuals develop complications typically associated with immunosuppression. In that case, a 35-year-old previously healthy male patient presented with nine days of diarrhea, malaise, and night sweats. He had a fever of 39.5°C, and both his abdominal exam and initial sigmoidoscopy were unremarkable, leading to a preliminary diagnosis of infectious diarrhea [11]. During the course of his illness, however, he developed retinitis, a complication that was ruled out in our patient. Stool cultures were negative, prompting a colonoscopy that revealed five ulcerated regions between the sigmoid colon and hepatic flexure. Histological analysis confirmed CMV, and the patient was subsequently treated with foscarnet.

What makes our case particularly notable is the presence of a colonic stricture, an uncommon complication in immunocompetent individuals, though it has been documented in immunosuppressed patients. One reported case describes a six-year-old boy with chronic granulomatous disease who underwent two bone marrow transplants and subsequently developed months of bloody diarrhea, fever, and abdominal pain. He was later found to have a focal colonic stricture at the splenic flexure, which was resected and revealed ulcerations consistent with CMV [12]. Another case involves a 77-year-old man with cirrhosis due to hepatitis C who had undergone liver transplantation. Ten years post-transplant, flexible sigmoidoscopy revealed an ulcerated stricture in the sigmoid colon, and biopsy confirmed CMV colitis. Similar to our patient, he was treated with intravenous ganciclovir followed by outpatient oral valganciclovir [13].

Delay in diagnosis contributes to a delay in targeted therapy for CMV colitis, leading to a more severe disease presentation, which is a common thread in the reported cases in immunocompetent patients. In addition to the aforementioned clinical aspects, socioeconomic factors may also play a role in the infection of immunocompetent patients. Colugnati et al. have shown that the force of infection of CMV colitis is higher in individuals of Latino descent, specifically Mexican-American and Non-Hispanic Black patients, as categorized in the study. In addition to these high-risk racial and ethnic groups, low socioeconomic background was also identified as a risk factor for CMV infection [14]. Our patient identifies as Latina ethnicity, which may have played a role in the occurrence of CMV colitis. Additionally, the patient’s primary language was Spanish and required an interpreter. While ethnicity may not contribute directly to a delay in the diagnosis, it is important to recognize the complexity added in providing and receiving medical care in a language other than the primary language spoken by providers. It has been shown that language barriers contribute to the length of healthcare visits and medical costs [15]. In the case of our patient, these factors could have contributed to the delay in diagnosis after the first presentation at the outside hospital emergency department.

## Conclusions

In summary, CMV-related colonic complications can include stricture formation, even in immunocompetent individuals. Maintaining a broad differential diagnosis is essential in patients with abdominal pain and colonic abnormalities, particularly when ulceration or granulation tissue is present on endoscopy or biopsy. In these settings, CMV immunohistochemistry should be considered on colonic biopsy specimens regardless of immune status, as tissue diagnosis remains the diagnostic cornerstone and routine histology alone may be insufficient. Early recognition of gastrointestinal CMV disease may reduce diagnostic uncertainty and help avoid unnecessary surgical intervention. In cases with stricture or mass-like lesions, repeat colonoscopy after antiviral therapy may also help assess mucosal healing and determine whether the stricture has resolved.
